# Supplementary figures and images for: EHMT2 methyltransferase governs cell identity in the lung and is required for KRAS G12D tumor development and propagation
Source: eLife. 2022 Aug 19;11:e57648. doi: 10.7554/eLife.57648 (PMC9439681; doi:10.7554/eLife.57648)

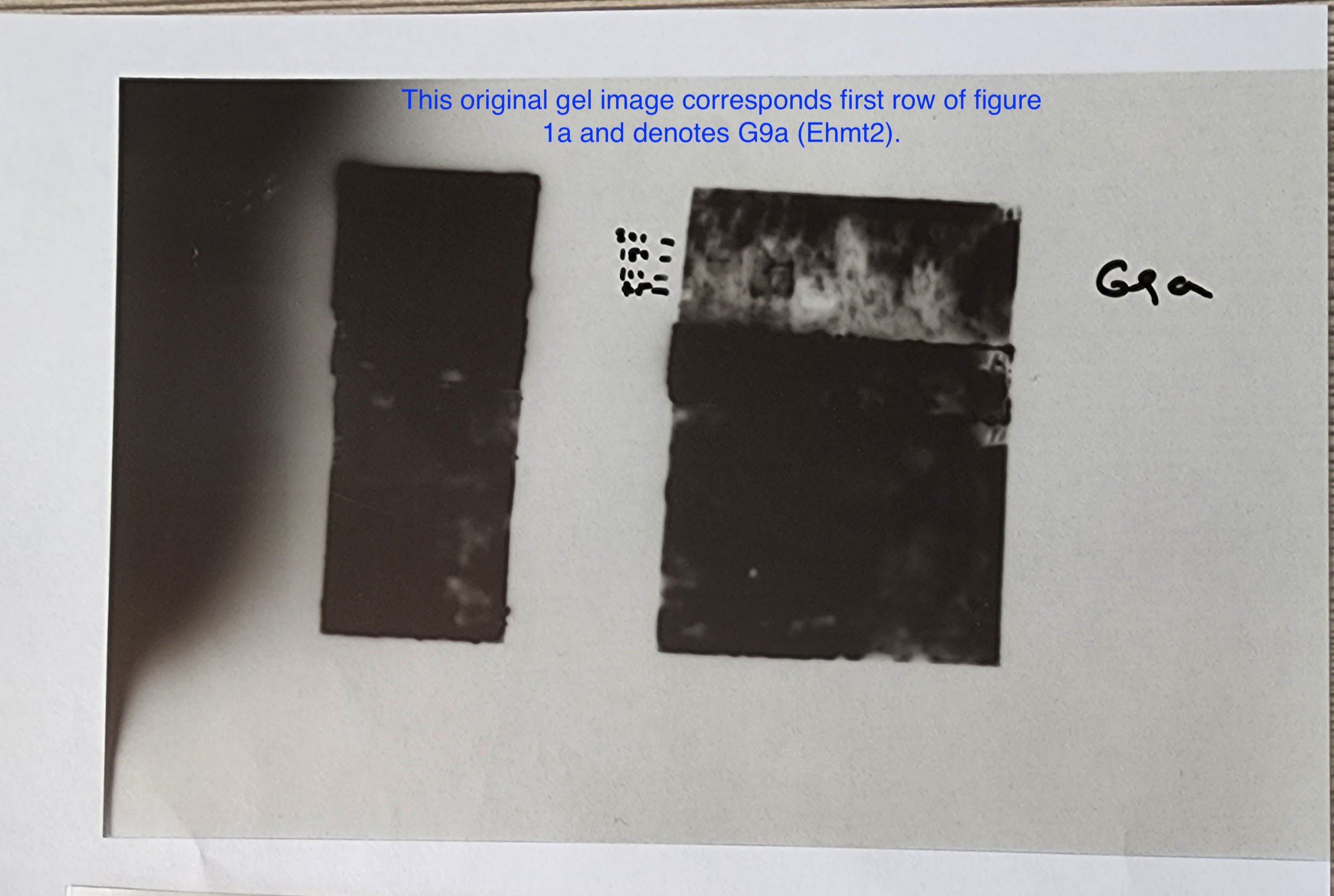

Supplement: Figure 1—source data 1. [file elife-57648-fig1-data1.zip › Figure 1 source data1 (2).jpg]

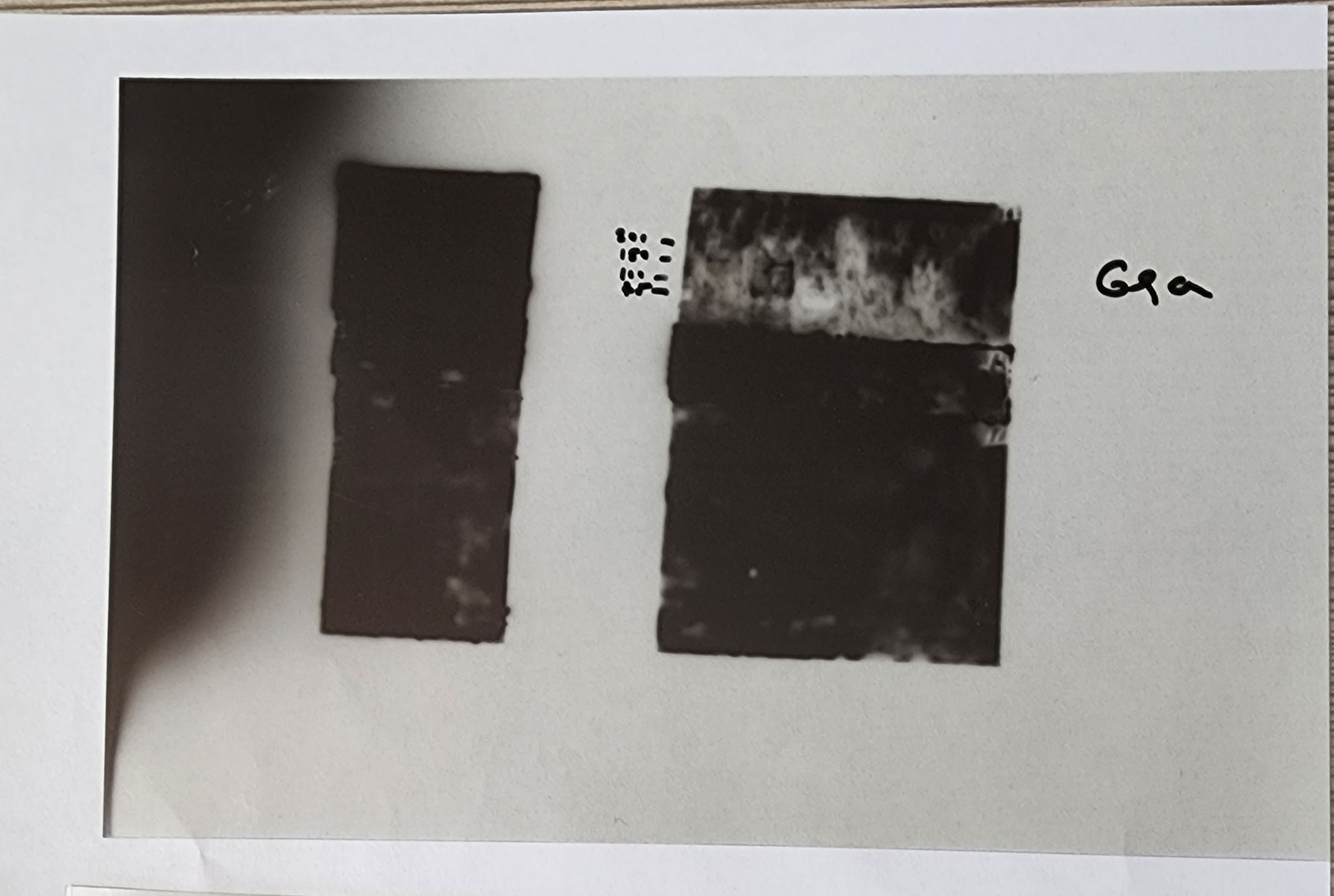

Supplement: Figure 1—source data 1. [file elife-57648-fig1-data1.zip › Figure 1 source data1 57648.jpg]

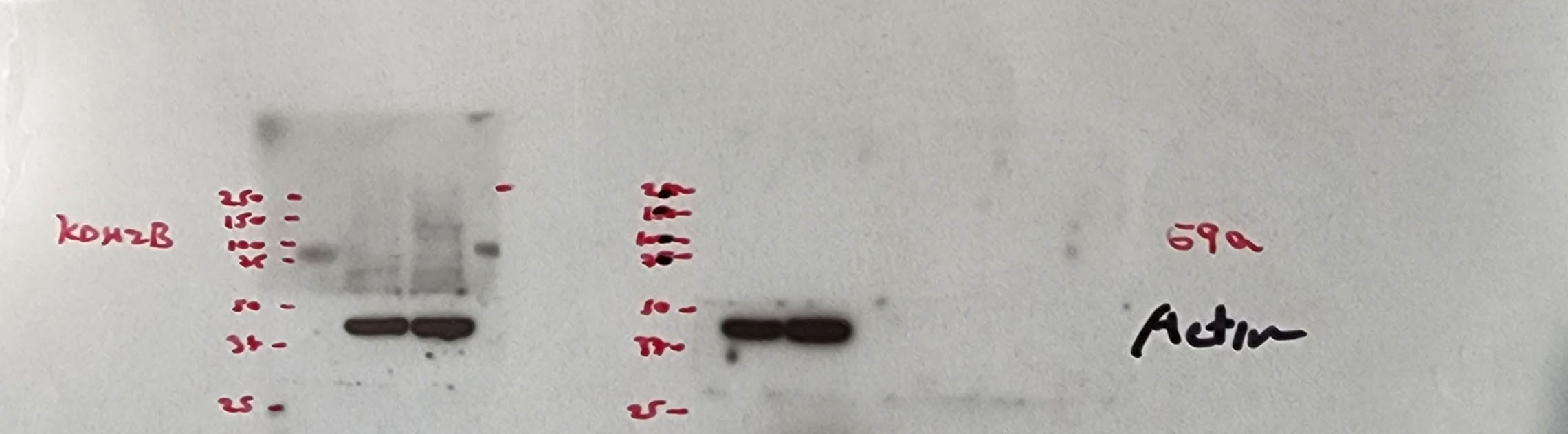

Supplement: Figure 1—source data 2. [file elife-57648-fig1-data2.zip › Figure 1 source data2 57648.jpg]

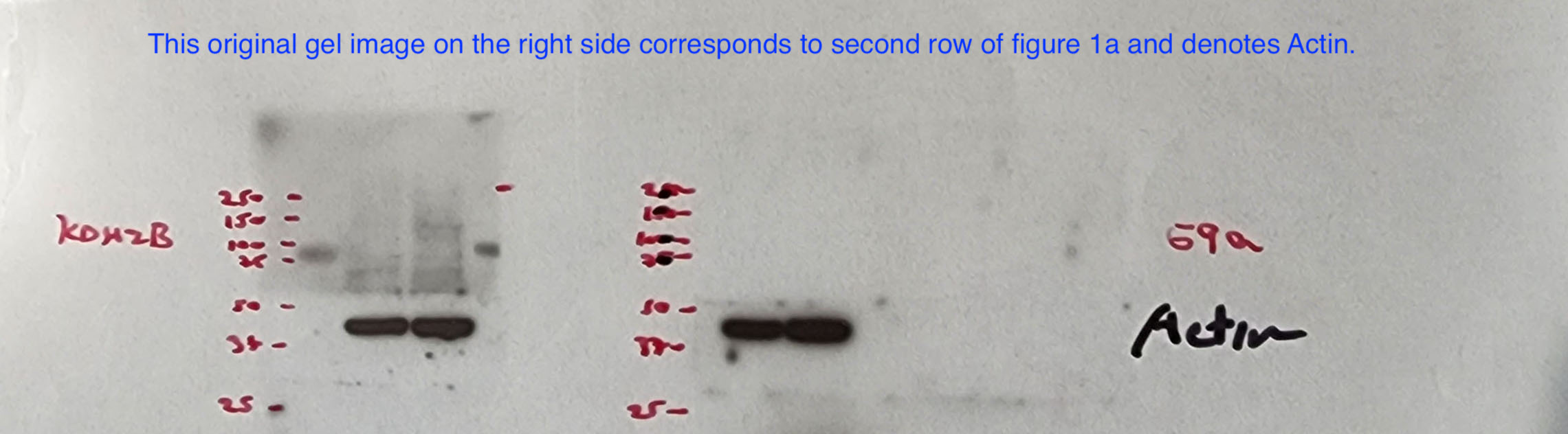

Supplement: Figure 1—source data 2. [file elife-57648-fig1-data2.zip › Figure 1 source data2 (2).jpg]

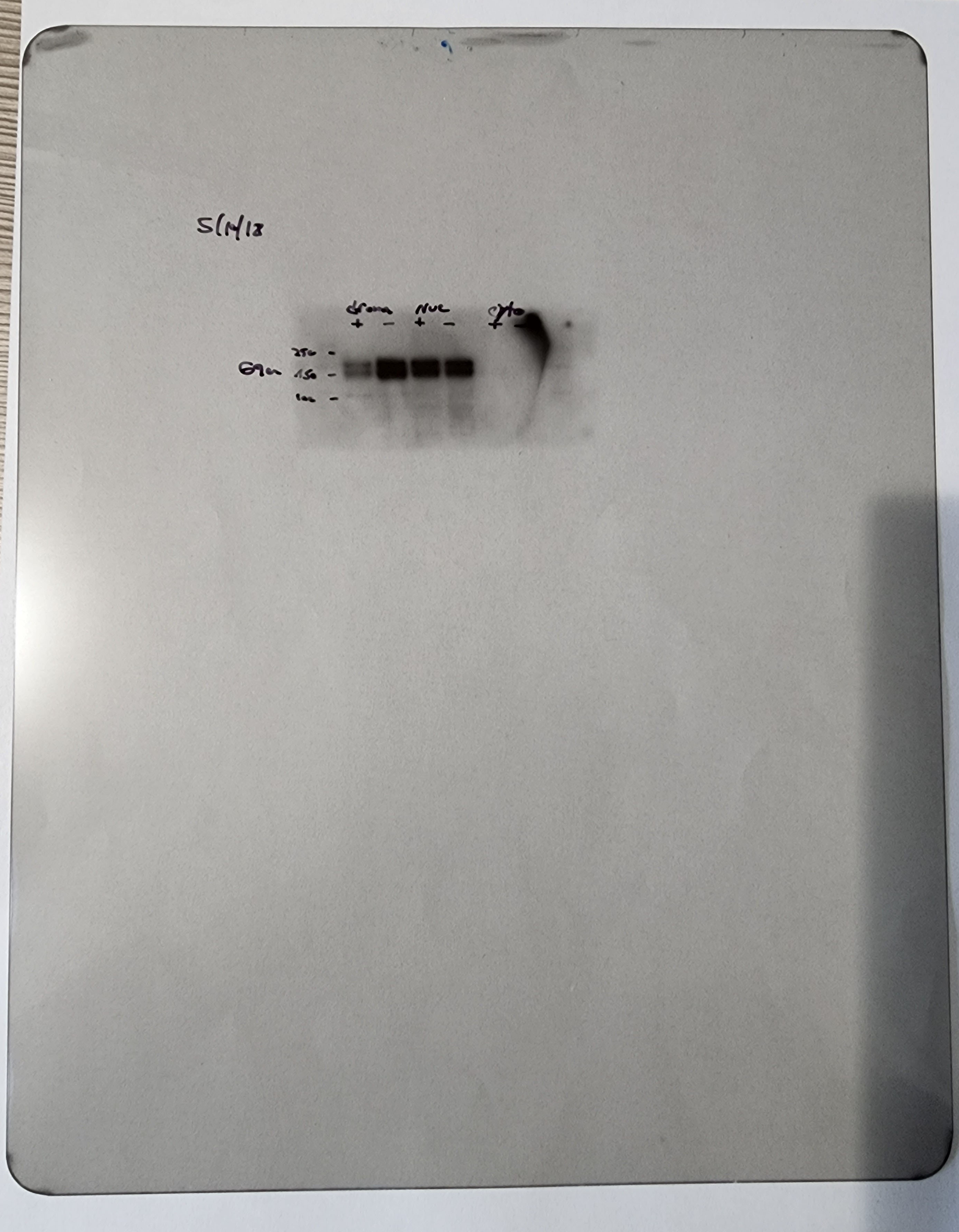

Supplement: Figure 5—source data 1. [file elife-57648-fig5-data1.zip › Figure 5 source data1 57648.jpg]

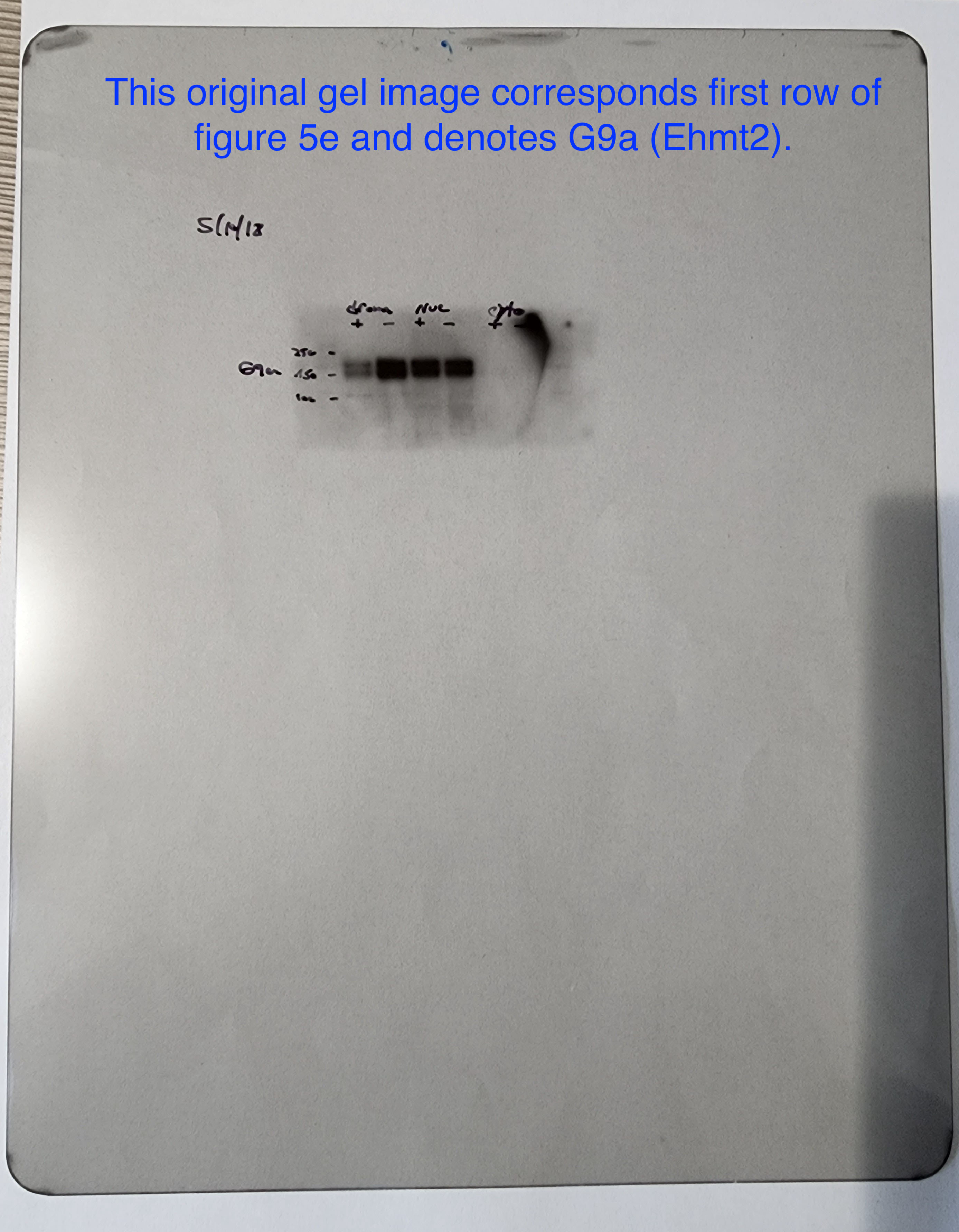

Supplement: Figure 5—source data 1. [file elife-57648-fig5-data1.zip › Figure 5 source data1 (1).jpg]

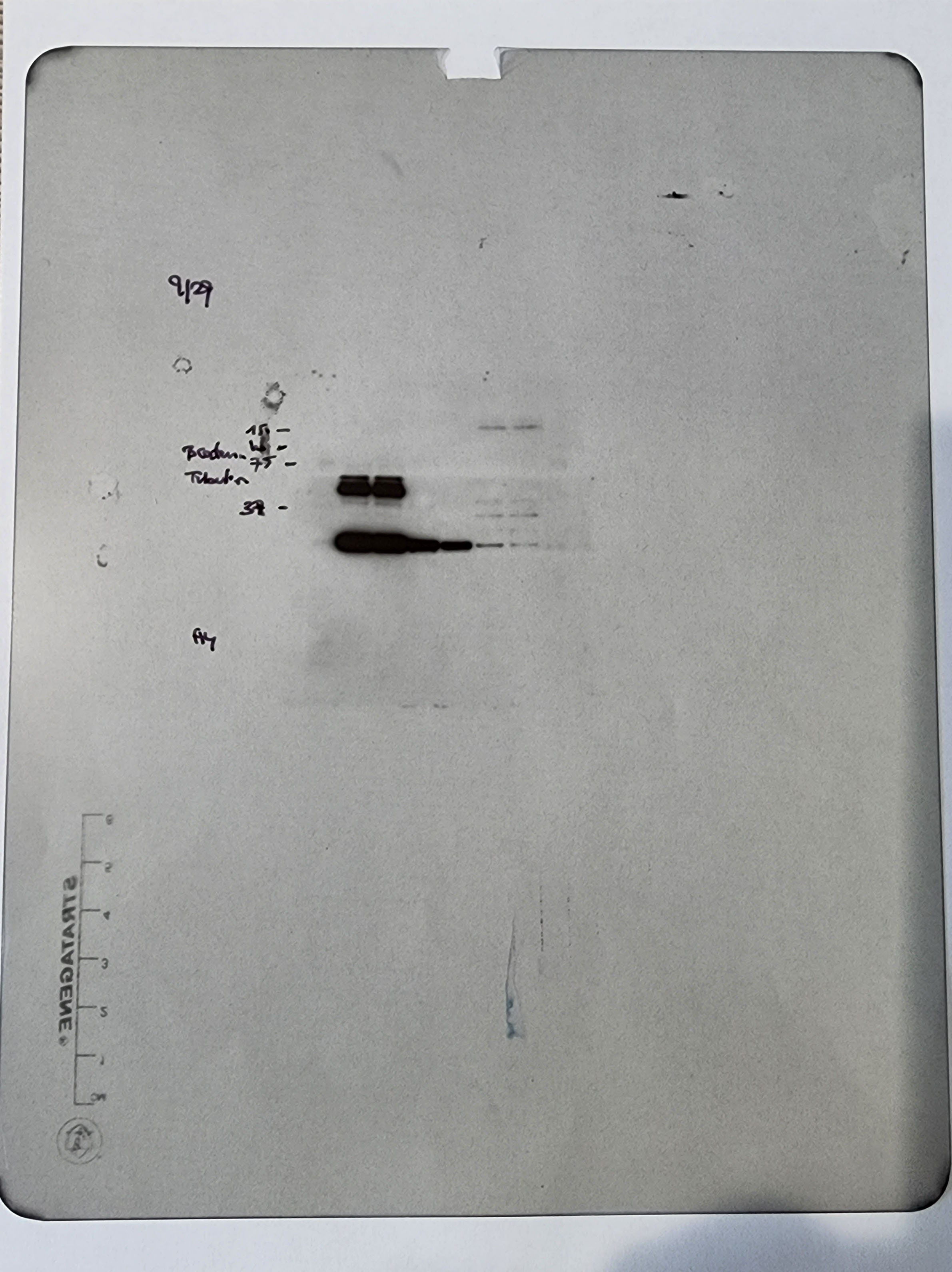

Supplement: Figure 5—source data 2. [file elife-57648-fig5-data2.zip › Figure 5 source data2 57648.jpg]

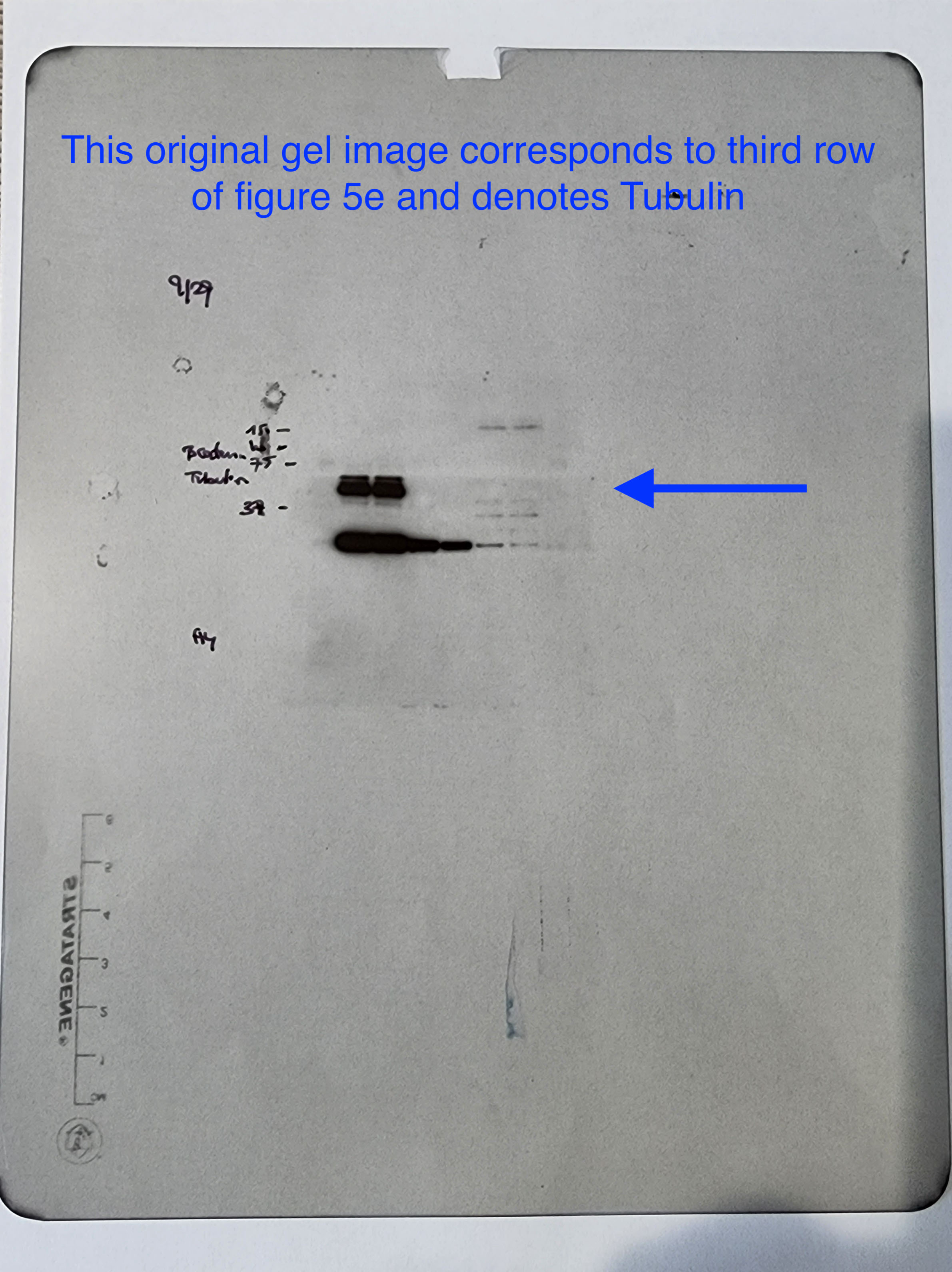

Supplement: Figure 5—source data 2. [file elife-57648-fig5-data2.zip › Figure 5 source data2 (1).jpg]

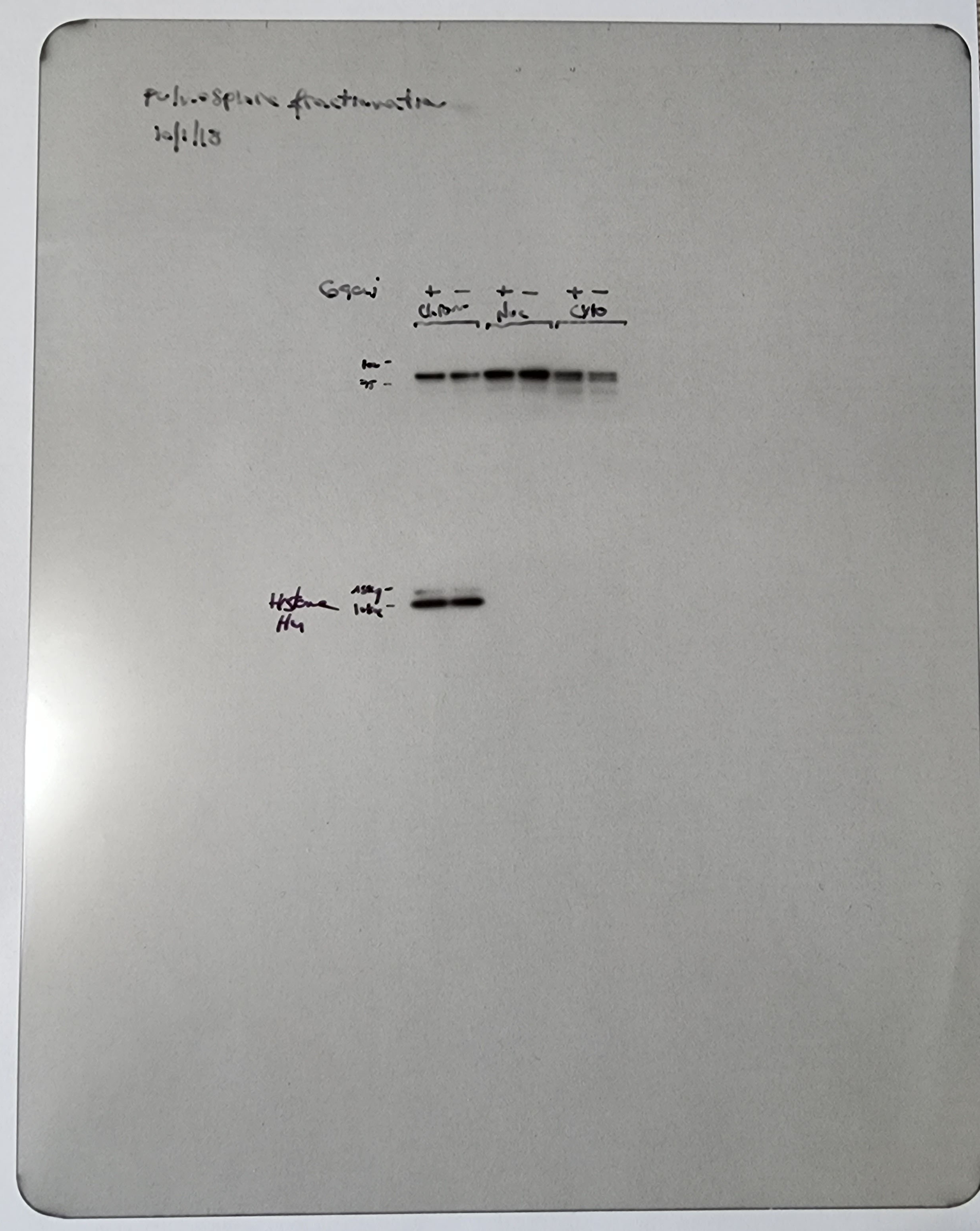

Supplement: Figure 5—source data 3. [file elife-57648-fig5-data3.zip › Figure 5 source data3 57648.jpg]

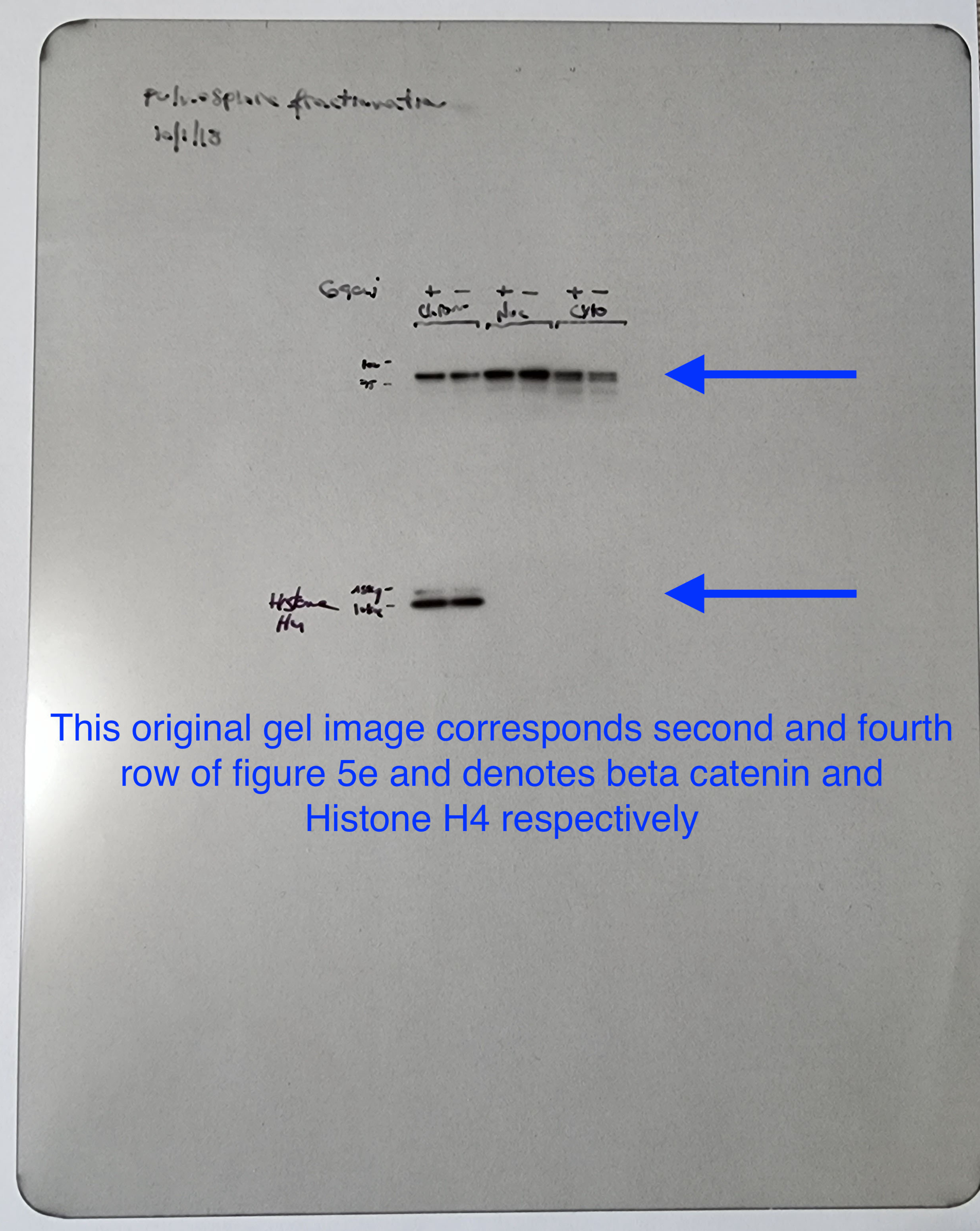

Supplement: Figure 5—source data 3. [file elife-57648-fig5-data3.zip › Figure 5 source data3 (1).jpg]

**
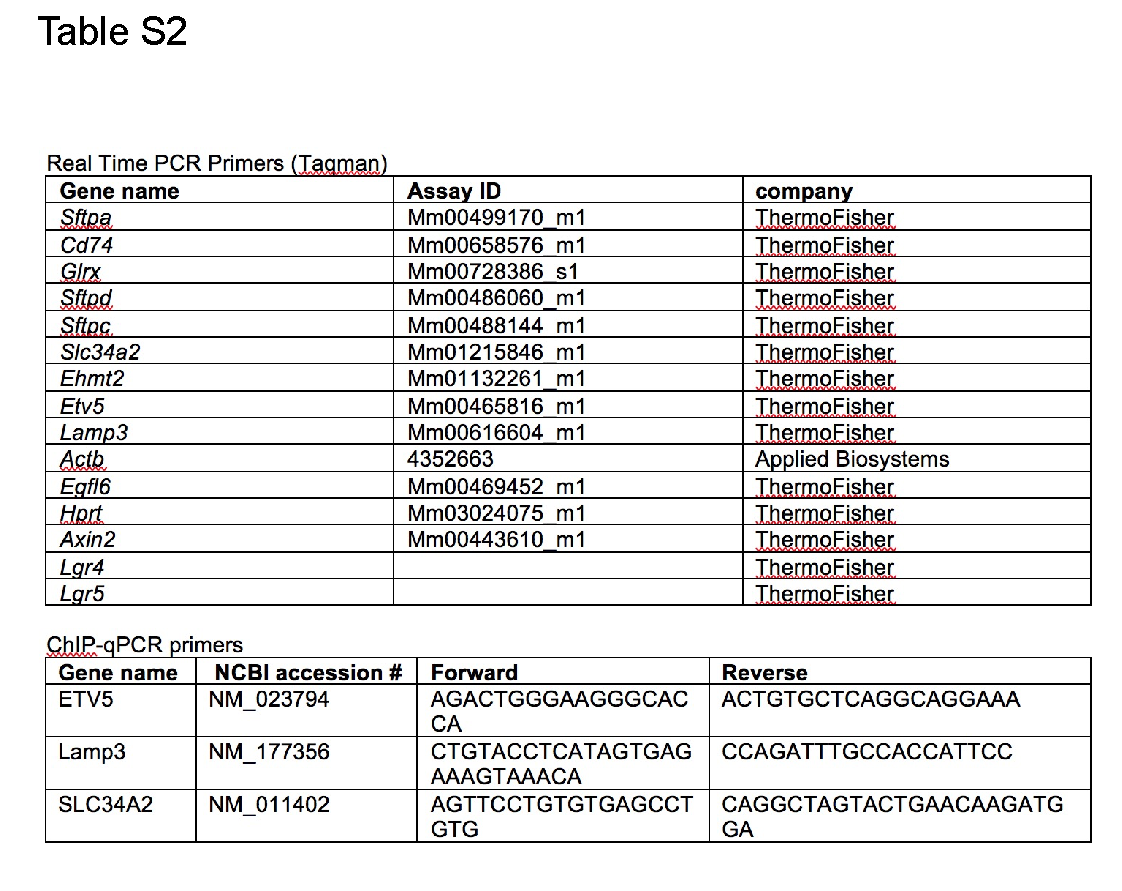
**

Supplement: Supplementary file 2. [file elife-57648-supp2.docx]
