# Supplementary material for: EHMT2 methyltransferase governs cell identity in the lung and is required for KRAS G12D tumor development and propagation
Source: eLife. 2022 Aug 19;11:e57648. doi: 10.7554/eLife.57648 (PMC9439681; doi:10.7554/eLife.57648)
Supplement: Supplementary file 1. — The seed for shEhmt2 was queried in NCBI BLAST and the resulting query results are shown. [file elife-57648-supp1.docx]

Supplementary Information for

G9a methyltransferase governs cell identity in the lung and is required for KRAS G12D lung tumor development and propagation.

Ariel Pribluda^1^, Anneleen Daemen^2^, Anthony Lima^1^, Xi Wang^1^, Marc Hafner^2^, Chungkee Poon^3^, Zora Modrusan^4^, Anand Kumar Katakam^5^, Oded Foreman^5^, Jeffrey Eastham^5^, Jefferey Hung^5^, Benjamin Haley^4^, Julia T Garcia^6^, Erica L. Jackson^7^ and Melissa R. Junttila^1^.

Departments of ^1^Translational Oncology, ^2^Bioinformatics & Computational Biology,

^3^Immunology, ^4^Molecular Biology, ^5^Pathology, Genentech, 1 DNA Way South San Francisco, CA 94080, USA. ^6^Department of Genetics, Center of Personal Dynamic Regulomes, Stanford, CA, 94305, USA. ^7^Scorpion Therapeutics, 2 Tower Place, 3rd floor, South San Francisco, CA 94080 USA.

*Current affiliation: Surrozen 171 Oyster point Blvd, #400, South San Francisco, CA, 94080

†Current affiliation: ORIC Pharmaceuticals 240 E. Grand Ave. South San Francisco, CA 94080

Corresponding authors: melissa.junttila@oricpharma.com, ariel@surrozen.com

**This file includes:**

Supplementary File 1

**
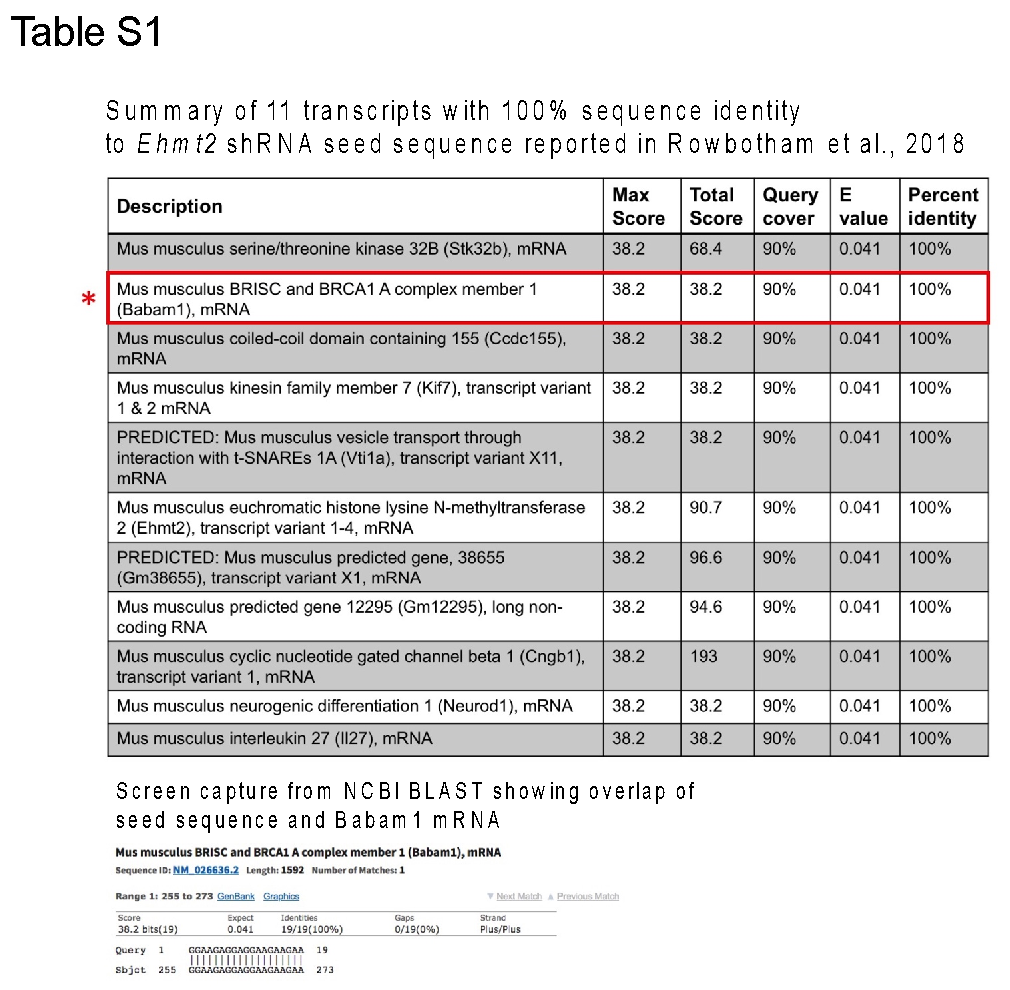
**
